# 

**Published:** 1886-04

**Authors:** 


### Illinois State Dental Society.

The twenty-second annual meeting will be held at Rock Island, Ill., beginning Tuesday, May 11th, 1886, and continuing five days.

Dentists in this and adjoining States are cordially invited to attend. J. W. WASSALL, Secretary,
208 Dearborn Avenue, Chicago.

### Kansas State Dental Association.

The fifteenth annual meeting of the Kansas State Association will be held in Topeka on Tuesday, May 4th, and continue three days. This meeting will be made the most interesting and profitable one in the history of the Association.

Members of the profession in other States are cordially invited to be present. Topeka is easy of access, and has excellent hotel accommodations. C. B. Bird, Sect., Topeka.

### THE DENTAL REGISTER,

A MONTHLY MAGAZINE DEVOTED TO THE INTERESTS OF THE

**DENTAL PROFESSION.**

*Terms Reduced to* $2.00 *Per Year. Single Copies,* 25 *Cents.*

**EDITED BY PROF. J. TAFT, D.D.S.**

—AND—

**Published by SAM'L A. CROCKER & CO., Ohio Dental and Surgical Depot.**

The volume commences in January and closes in December.

The REGISTER being issued monthly is always fresh, therefore Indispensable to the practitioner who desires to keep posted up with the new inventions and improvements which are daily developed. Neither expense nor effort will be spared to give value and variety to its contents. Articles will be illustrated with well executed engravings whenever they will add to the interest or assist to explanation.

Its extensive circulation and frequency of issue make it one of the BEST DENTAL ADVERTISING MEDIUMS in the United States.

All subscriptions must begin with January or July.

Local or special notices, five lines or less, will be inserted for $3 each insertion; over five lines, 50 cts. per line.

All advertising bills payable in advance, or at furthest, after the issue of the first number containing the advertisement. All transient advertisements to be paid for in advance.

☞CONTRIBUTIONS SOLICITED.

Exclusively Editorial Communications should be addressed to the Editor.

The latest number of the REGISTER may be ordered with or without other goods at any time, and all letters should be addressed to

**SAM'L A. CROCKER & CO., Ohio Dental and Surgical Depot, Cincinnati, O.**

